# Piecing together systematic reviews and other evidence syntheses: a guide for librarians

**DOI:** 10.29173/jchla29706

**Published:** 2023-08-01

**Authors:** Chelsea Misquith

**Affiliations:** Librarian Toronto, Ontario, Canada

Foster MJ, Jewell ST, editors. **Piecing together systematic reviews and other evidence syntheses: a guide for librarians**. Lanham, MD: Rowman & Littlefield; 2022. Hardcover: 345 p. ISBN: 9781538150177. Price: USD $85.00. Available from: https://rowman.com/ISBN/9781538150184/Piecing-Together-Systematic-Reviews-and-Other-Evidence-Syntheses.

As any librarian who works closely with health sciences researchers is aware, systematic reviews and other evidence syntheses are a rapidly-growing area of research support requests today. Over the past few years, evidence syntheses have also been growing in popularity in fields such as economics, education, psychology, and the social sciences. Given this context, *Piecing together systematic reviews and other evidence syntheses: a guide for librarians (2022)* could not have been published at a better time. This book is a follow-up to the editors’ previous book, *Assembling the pieces of a systematic review: a guide for librarians*, published in 2017. As mentioned in the preface, *Piecing together* expands on the first book and is also geared towards librarians who are closely involved in supporting evidence syntheses in any of the following ways: providing consultations and workshops on the topic, contributing as co-authors, or managing review support services at their libraries.

Each chapter of the book is written by librarians who are widely considered to be experts in evidence syntheses, with many years of experience under their belts. Many of them have actively contributed to the development of guidelines and standards for librarians and non-librarian researchers who are interested in working on evidence synthesis projects. The editors of the book are themselves prolific in this regard: Foster is a Professor at Texas A&M University and Director and Founder of the Center for Systematic Reviews and Evidence Syntheses at Texas A&M. Jewell is Assistant Director of Clinical Services at Weill Cornell Medicine and launched the systematic review service at Memorial Sloan Kettering Cancer Center Library. In addition to the editing the book, Foster and Jewell have both co-authored a few of the chapters.

The book comprises 22 chapters that have been divided into 7 sections: (i) the big picture, (ii) plan, (iii) identify, (iv) evaluate, (v) collect, combine, and explain, (vi) summarize and share, (vii) the art of puzzle solving. The chapters in section (i), the big picture, cover the differences between the most common evidence syntheses and introduce readers to the PIECESS Framework. As outlined in chapter 1, “the PIECESS Framework is designed to detail the complexity of systematic, scoping, mapping, and other research projects”. The Framework comprises 8 phases (proposal of scope, protocol registration, preliminary findings, paper completion, preservation of project, promotion to stakeholders, impact compilation, update review) that works through 8 processes that form the acronym PIECESS: Plan, Identify, Evaluate, Collect, Combine, Explain, Summarize, and Share. As readers will note, the next 5 sections in the book are structured following the PIECESS Framework. The last section covers information relevant to developing and managing a systematic review support service at a library.

The book is organized in a way that makes it easily digestible from start to finish, and also allows readers to become familiar with the PIECESS Framework. It is structured cohesively and flows through the natural order of tasks that are involved in evidence synthesis projects, all the way from picking a research question (and framing it appropriately) to deciding how to store and share the associated data that is created during the project. Each chapter has references listed at the end of the chapter which allows easy access for the reader. Additionally, each chapter also has tables, checklists, comparison charts, and other important resources that make it easy for readers to go back to if they need to refresh their memory. For example, chapter 6 has a chart (Box 6.4) comparing select features among the most common systematic review software programs. This chart would be helpful for librarians who want to recommend a software for purchase at their library, or who are asked by researchers to recommend a software for use for their project. The writing is easy to follow and any jargon or acronyms are defined and expanded upon.

There were a few aspects of the book that especially stood out to me. I appreciated the inclusion of case studies of searches for a few common types of evidence synthesis projects (chapter 10). They demonstrate what searches and documentation should look like and why they should look this way. I definitely could have benefited from reading something like this early on in my career! I also appreciate the last section of the book, the art of puzzle solving, as there is a wealth of information about organizing and managing new and existing evidence synthesis support services. Chapter 18 delves into the most common types of review services at libraries today and emphasizes the importance of assessing review services. That said, the section would be a great read for library administrators, librarians who are responsible for managing review support services at their libraries, or any librarian who wants to advocate for more funding, resources, staffing, and organizational support to develop and manage review support services at their library.

In conclusion, this book serves as a comprehensive reference on evidence synthesis support services in libraries and provides an absolute wealth of information on the topic. I would recommend this book for all librarians who either currently support evidence syntheses as part of their job, or plan to do so in the future. Looking back, I could have used a book like this in previous jobs and on previous evidence synthesis projects, and will most definitely keep it as a reference to consult in the future! I would also recommend this book for library administrators, especially those who have not worked on evidence synthesis projects themselves. By reading it, they might gain a better understanding of the level of effort, time, and skill that goes into these projects. They could review recommendations and comparison charts for software and other tools. Finally, I would also recommend that librarians share this book as a resource with non-librarian researchers they are working with. While non-librarians are not the editors’ intended audience for the book, it could be useful as a teaching tool during research consultations and workshops.

